# A Cold Plasma Technology for Ensuring the Microbiological Safety and Quality of Foods

**DOI:** 10.1007/s12393-022-09316-0

**Published:** 2022-06-24

**Authors:** Ozioma Forstinus Nwabor, Helen Onyeaka, Taghi Miri, Kechrist Obileke, Christian Anumudu, Abarasi Hart

**Affiliations:** 1 Division of Infectious Diseases, Department of Internal Medicine, Faculty of Medicine, Prince of Songkla University, Hat Yai, Songkhla, 90112 Thailand; 2 School of Chemical Engineering, University of Birmingham, Edgbaston, B15 2TT UK; 3 Renewable and Sustainable Energy, University of Fort Hare, Alice, 5700 Eastern Cape South Africa; 4 Department of Chemical and Biological Engineering, The University of Sheffield, Sheffield, S1 3JD UK

**Keywords:** Cold plasma, Food safety, Shelf-life, Microbial spores, Food quality

## Abstract

Changing consumers’ taste for chemical and thermally processed food and preference for perceived healthier minimally processed alternatives is a challenge to food industry. At present, several technologies have found usefulness as choice methods for ensuring that processed food remains unaltered while guaranteeing maximum safety and protection of consumers. However, the effectiveness of most green technology is limited due to the formation of resistant spores by certain foodborne microorganisms and the production of toxins. Cold plasma, a recent technology, has shown commendable superiority at both spore inactivation and enzymes and toxin deactivation. However, the exact mechanism behind the efficiency of cold plasma has remained unclear. In order to further optimize and apply cold plasma treatment in food processing, it is crucial to understand these mechanisms and possible factors that might limit or enhance their effectiveness and outcomes. As a novel non-thermal technology, cold plasma has emerged as a means to ensure the microbiological safety of food. Furthermore, this review presents the different design configurations for cold plasma applications, analysis the mechanisms of microbial spore and biofilm inactivation, and examines the impact of cold plasma on food compositional, organoleptic, and nutritional quality.

## Introduction

Food preservation is an age-long science that has continuously evolved. Ancient people employed physical means, including sun drying, roasting, smoking, fermentation, and salting, to preserve farm produce. With the advent of science and technology, chemicals with antimicrobial and antioxidant properties were adopted as ideal food preservatives. However, contemporary consumers’ taste, guided by scientific revelations on the adverse health impact of most food preservatives, has resulted in a craving for minimal preserved food, considered healthy and safe. Synthetic preservatives including butylated hydroxytoluene, butylated hydroxyanisole, sorbic acid, propyl gallate, and sodium nitrate have been correlated with cytotoxicity, suppression of immune response, and genotoxicity [1–3]. Thus, to accommodate the trending consumer’s taste, and ensure consumers’ safety, several advances in food science and technology have emerged as an alternative, effective, and safe approach for preserving food products and agricultural produce [4–6]. Also, the use of multiple combinations of food preservation techniques is suggested to enhance the safety of food products while ensuring extended product shelf-life [7, 8]. Unfortunately, high demands are placed on the ideal preservation technique, with several lofty expectations. The acceptance of preservative technique depends on factors ranging from health effects, cost and energy demands, duration of the shelf-life extension, ability to inactivate foodborne pathogenic and spoilage vegetative microorganisms and spores, effects on food nutritional and organoleptic properties, timing, and adaptability. Food preservation is a sacrosanct component of the food production chain that promotes food security. Current estimates suggest that one-third of agricultural produce are lost to food spoilage, with 25% of the total food loss attributed to microbial spoilage [9]. Microbial-mediated food deterioration is likely the most frequent cause of food loss and wastage of agricultural produce. The lack of a proper preservative/storage facility contributes significantly to the global food security crisis. Hence, proper storage and food preservation are key agricultural goals to ensure food security and availability. Although gaint progressive strides have been made in the science of food preservation and processing, the goal of achieving a natural, unaltered food with maximum safety remains elusive. Microbial harzards including food spoilage and foodborne diseases and food contamination demand greater investments towards ensuring consumers’ safety.

Food safety is a critical global issue that requires the concerted efforts of both food producers and consumers. The spread of pathogenic microorganisms, microbial toxins, and other contaminants through food and drinks is a major disease and infection source. Mortality and morbidity due to foodborne diseases and intoxication are the major sources of economic loss. Although the primary aim of preservation is the extension of food shelf-life, by extension, preservation ensures the safety of food products through the controls of pathogenic foodborne microorganisms. Various chemical food preservatives possess antimicrobial properties that inhibit the proliferation of spoilage and pathogenic microorganisms.

Similarly, most evolving green technologies employed to preserve food inhibits microbial growth and, at increased intensity, inactivates microbial cells. Although most preservation and storage techniques such as pasteurization and freezing can effectively inhibit or inactivate foodborne microorganisms, the presence of microbial spores in food constitutes a significant risk to food safety. Under preservation treatments and unfavorable storage conditions, certain pathogenic foodborne bacteria belonging to the genus *Clostridium* and *Bacillus* adopt the inactive spore forms to survive. The subsequent regermination of spores into vegetative cells leads to food contamination, especially of finished products. Microbial spores are recalcitrant to food processing and preservation treatments and are frequently associated with contaminated of finished processed products. Effective inactivation of microbial spores is a fundamental requirement for an ideal food preservation regimen. Several emerging technologies, although capable of effectively inactivating microbial vegetative cells, fail to eliminate spores. Microbial spores survive treatments such as thawing, heating [10], freezing, and UV radiations [11–13]. The presence of microbial spores in food products is a major cause of foodborne disease and intoxication. Thus, several novel technologies have been proposed to address challenges posed by the spore forming pathogenic foodborne microorganisms. Some of these methods have been reported to inactivate microbial spores in food, food processing facilities, and contact surfaces. Treatment of food with pulse electric field [14, 15], plasma technology [16, 17], ultrasonication [18–20], and high hydrostatic pressure [21] alone or in combinations demonstrated promising results for spore inactivation in food products.

Moreover, cold plasma must be evaluated critically as an emerging food processing alternative before being accepted by the food industry. In 2018, Pankaj et al. [22] published a review of cold plasma technology that discussed the negative effects and limitations. The overview did not explain how spore-forming microbes and spores are inactivated. Recently, Laroque et al. [23] published another review that describes in detail the most promising cold plasma sources and operation modes, the influence of operating conditions on plasma properties, and the efficacy of this technology in food processing. This review looked at the design of cold plasma processes that inactivate target microorganisms with minimal impact on food quality. The previous review papers examined the impact of cold plasma species on food components at molecular level and design methods to minimize the impact of the interactions. A review on the effects of cold plasma on the chemical structure of different food components and their impact on food attributes has been reported by Saremnezhad et al. [24]. But this review aims to highlight cold plasma technique as a recent ideal green preservation technique by comprehensively elucidating its benefits/advantages, superior attributes at spore inactivation, mechanism of microbial inactivation, and factors that should be considered to ensure optimal outcomes. Conversely, the formation of biofilm on food contact surfaces is a critical safety concern in the food industry, since it is a potential source of contamination; for this reason, the most distinctive contribution of this review focuses on exploring how biofilm can be disrupted through plasma treatment. The objective is to provide food researchers, technologist/engineers, and industrialist with additional knowledge that could help promote and ensure effective understanding and application of cold plasma technique in food preservation and for enhancement of food safety.

## Plasma Technology

In the food industry, several green technologies have been adopted to rid production lines and products of microorganisms that could compromise product quality, resulting in recalls and foodborne illness outbreaks. This has led to an upsurge on the topic of applications of green technologies in preservation and shelf-life extension. Over the last decade, the use of plasma has gained extensive applicability in the food industry as a relatively new and promising non-thermal decontamination technology. The technology is not limited to the food industry alone but extends to other sectors, including the surface decontamination of medical devices and environments [25–28], sanitization of heat-sensitive biological and chemical agents [29], environmental degradation of waste and toxic residues [30], and in the textile industry [31–33]. Its technique involves the application of plasma such as ionized or partially ionized gases to inactivate food contaminants, including microbial cells, enzymes, and toxins. The plasma is created either by sufficiently heating gas in an enclosed chamber under deep vacuum or by using radiofrequency or microwave energy to excite the gas molecules in order to produce free radicals, which are the main constituents of plasma.

Plasma has been described as the fourth state of matter (i.e., solid, liquid, gas, and plasma). It is a collection of both neutral and charged particles referred to as reactive species. In a plasma state, molecules dissociate into their atomic elements, losing electrons and acquiring a higher energy state [34]. The efficacy of plasma technology depends on factors such as energy defined as pressure, temperature, the thermodynamic equilibrium between the particles [34], and the type of gas used [35]. Though plasma induction requires sufficiently high energy, recent advancements in plasma physics have made it possible to generate “cold plasma” at ambient temperatures and atmospheric pressures. This generated cold plasma can be applied in medicine for sterilization [26], wound healing [36], and disease treatment [37, 38]; in agriculture to enhance seed germination [39–41]; and in the food industry for inactivation of vegetative and spores of foodborne pathogenic and spoilage microorganisms, enzyme inactivation, and toxin inactivation. Consequently, cold plasma has also been employed in environmental management for the degradation of contaminants such as pesticides [42] and dyes [43], and for the decontamination and treatment of wastewater [44–47]. Other areas of applicability include catalysis and material sciences for surface modification and functionalization [48–50] as well as sterilization. However, the application of cold plasma in food sterilization has revolutionized preservation technology, offering a healthy alternative with negligible effect on the food nutritional and organoleptic properties. The cold plasma is produced through partial ionization of gaseous molecules [51]. For food processing, plasma generation methods are classified as dielectric barrier discharge (DBD), plasma jet (PJ), corona discharge (CD), radiofrequency (RF), and microwave (MW). A detailed review of the specifics and applicability for each has been reported by Laroque et al. [23]. Researchers are most interested in DBD plasma generation methods because they are a safe and low-cost alternative for processing applications. Applied electric fields and spatial charge distribution are responsible for electron energy, which drives plasmas’ reactivity, which is influenced by the chemical composition of the gas. However, plasma properties are influenced by the size, time scale, temperature, and density of the charged and reactive species [52]. In a recent review published by Laroque et al. [23], factors such as gas composition, relative humidity, and electrode and dielectric barrier including electrode and dielectric materials, electrode geometry, and shape have been discussed in relation to plasma reactive species formation and ionization, efficiency, and action.

**Fig. 1:**
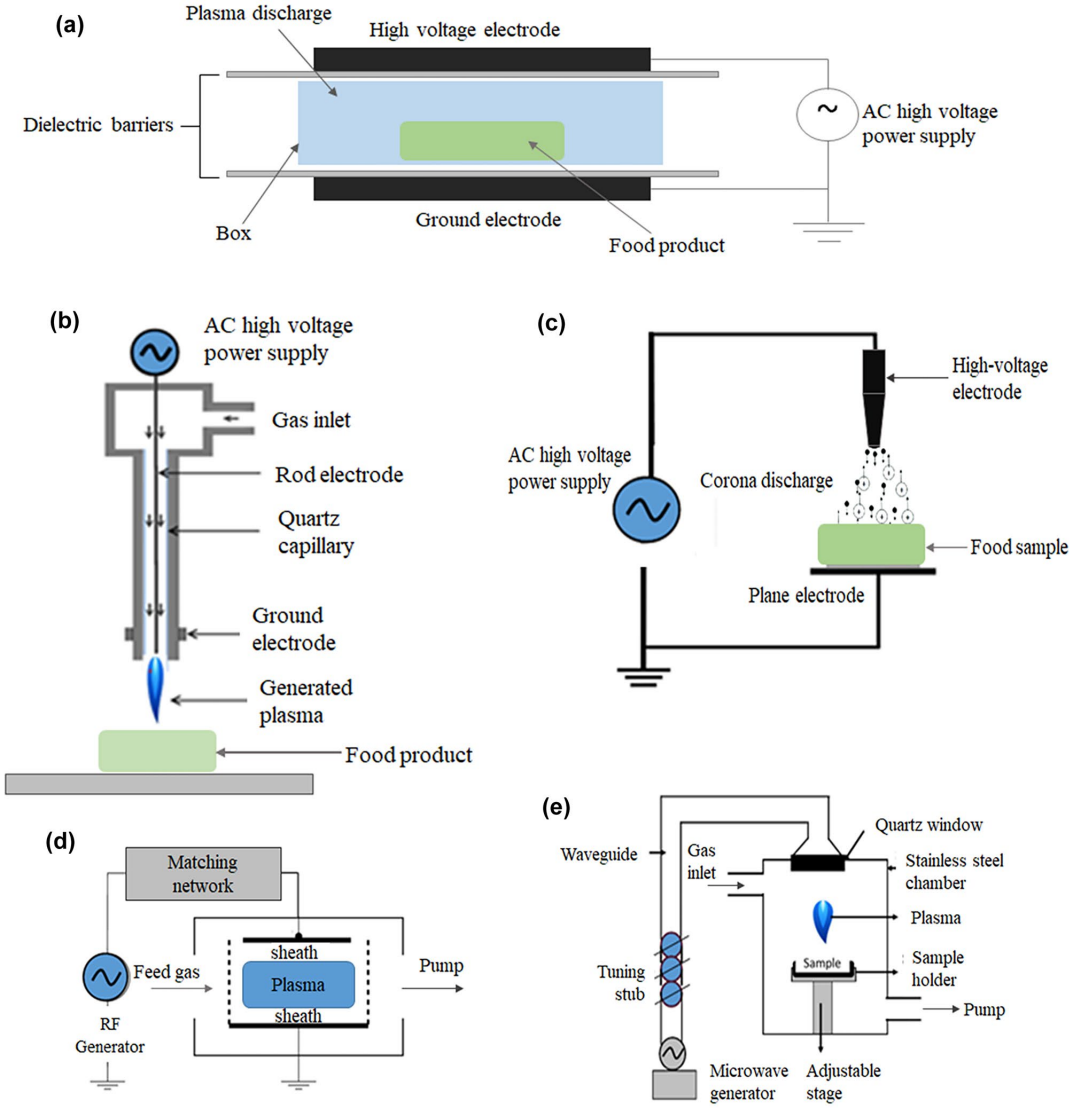
Different configuration of cold plasma designs for food safety applications: **a** dielectric barrier discharge, **b** plasma jet, **c** corona discharge, **d** radiofrequency, and **e** microwave plasma system

Processing food is an important step in extending the shelf life and preserving the nutritional quality of food while reducing post-harvest losses. With cold plasma, a gaseous matter is converted into an ionized gas known as plasma (comprising electrons, ions, neutral molecules, and atoms as well as charged reactive species) when sufficient energy is applied. The application of cold plasma technology for food safety and preservation can be categorized into three types as follows: (i) direct plasma treatment which is generated at the site of application, (ii) indirect plasma treatment in which it is created at a remote location and delivered to the target, and (iii) solution treated with plasma such as plasma-activated water used as a disinfectant [53]. The configuration of different cold plasma designs food safety applications is shown in Fig. 1. As shown in Fig. 1a, plasma can be generated via dielectric barrier discharge (DBD) by dispersing current flow through dielectric materials between electrodes. In packaging where reactive oxygen and nitrogen species can be generated directly within sealed packages, the DBD is highly appropriate for inactivating microorganisms on fresh produce. Typical operation conditions are gas pressure in the range of 10^4^–10^6^ Pa and frequency 10–50 MHz. Due to its cost-effectiveness, configuration flexibility enables food treatment in-package (such as meats, poultry, fruits, and vegetables) and prevents post-contamination, and consequently, the most convenient method of plasma generation, the DBD method, is the most commonly used [23, 54]. Figure 1b illustrates the design configuration of plasma jet in which an inlet gas flows between electrodes while the outer electrode is grounded; the central electrode is driven by high-voltage power supply to produce free electrons that collide with gas molecules to produce various reactive species. The discharge plasma is utilized for the treatment of the food product. However, the application is area-limited. Corona plasma discharge is classified as atmospheric pressure cold plasma. It occurs when current flows from an electrode with a high potential into a space filled with air or other gases, ionizing it in the process and creating a region of plasma around the electrode as shown in Fig. 1c. It can be powered with high-pulsed DC or AC voltage. Unlike the other electrode configurations, the electrodes here are highly asymmetrical, designed with a thin wire or needle electrode facing flat plane or large diameter cylinder electrode. Furthermore, corona discharge arcs are commonly created by strong electric fields generated by small diameter wires, needles, or sharp-edge electrode (see Fig. 1c). Notably, the corona plasma region occurs in the immediate locality of the of the point electrode. Unlike plasma jet, the corona plasma discharge has a more extensive coverage of food sample area and also produces denser and more energetic plasma compared to DBD [55]. However, the produced plasma is characteristically inhomogeneous, limiting its application for uniform treatment.

On the other hand, radiofrequency (RF) plasma is formed by applying a radio frequency field to a gas flow as shown in Fig. 1d. RF plasmas can be classified into capacitively coupled plasma, inductively coupled plasma and helicon wave sources. The design configuration consists of two parallel electrodes separated by a gap of a few centimeters in a vacuum chamber [23, 54]. Methodologically, it can be operated in the range of 1–100 MHz [56]. In contrast, microwave discharge is generated by a magnetron with a typical electromagnetic wave frequency of about 2.45 GHz (Fig. 1e). By directing the electromagnetic waves into the treatment chamber, the microwaves are absorbed by the gas which heats and ionizes, causing the release of electrons. This results in ionization reactions because of inelastic collisions, releasing energy as photons of visible light and UV light. Unlike other plasma source that uses electrodes, it uses antenna switches as microwave gas discharge plasma devices which uses the high-power microwave pulse for plasma generation [57]. The purpose of this pulse is to prevent damage to the receivers of low-signal microwaves through the circuit. The microwave electromagnetic field accelerates the electrons of gas molecules to create plasma. The technique has the potential to generate both quasi-equilibrium and non-equilibrium plasma for different applications [57]. Microwave plasma configuration comprises of the following components: a power source such as magnetron, circulator, standing wave ratio meter, matching circuit/network and microwave-to-plasma applicator.

### Cold Plasma Design

Three major designs of cold plasma technology for food sterilization are currently employed. These include remote treatment, direct treatment, and close proximity with an electrode. These designs are configured according to the positioning of the source that generates the plasma and the surface of the target [53]. In the remote treatment design, the target product is not placed directly in the plasma chamber. The ionized gas used for remote treatment might be air, nitrogen, or a mixture of noble gases [53]. The limitation of the remote treatment design is the generation of reactive species which might interact with other plasma species such as charged particles or photon species. However, in terms of design, the remote treatment is preferred over others because of its design simplicity, size flexibility, and target physical shape. Unlike the remote treatment, in the direct treatment, the target product is placed in direct contact with the generated plasma and often employs the plasma needle and microwave plasma tube [53]. The direct treatment results in higher exposure to UV radiation because of the closeness of the target product. This results in high moisture content due to the heat generation by conduction in the product [58]. This process alters the appearance and texture of food products such as meats and deteriorates vitamins and nutrients. In close proximity design, the food product is placed in close proximity to one of the electrodes. This type of design ensures that the target product is exposed to a higher combination of reactive charged particles, electrons that are negatively charged, and UV radiation [59]. For effective operation of this design, products to be treated or sterilized must fit between the electrode. The system can be best used for smaller food products such as seeds, berries, nuts, and flatter objects like chicken breast [53].

### Cold Plasma as an Ideal Preservation Method

Plasma processing uses cold plasma to extend the shelf-life of products and is a green, contemporary preservation method that is compatible with a range of food types. The efficacy of the technology has been demonstrated in several reported articles on a wide variety of food, including natural and processed products. Also, the application of the method to surface sanitization of in-package fruit and other food products, including vegetables, meat, cereal grains, has demonstrated promising results, with effective eradication of microorganisms, prolonging of products shelf life, reducing spoilage losses, and improving nutritional, functional, and sensory properties of food products [60]. In addition, cold plasma technology offers numerous advantages, including rapid processes, enhanced process efficiency, elimination of process steps, better quality product, and retention of product characteristics (e.g., texture, nutrition value, organoleptic properties), and improved shelf life. Table 1 highlights reported applications of cold plasma technology in different food types. It is clear that significant log reduction of spore-formers microbes as well as inactivation spores itself can be achieved with cold plasma technology. Also, the technique can be applied for a wide variety of purpose such as decontamination, inactivation of spores and spore-formers, food quality preservation, in-packaging treatment, and food self-life extension (Table 1). Consequently, it can be applied to food products in various forms: juice, vetegables and fruits, and meats. One of the major advantages of cold plasma processing over other non-thermal technologies is the evidenced effectiveness at spore inactivation [17, 61], and it also inactivates microbial toxins [17, 62, 63]. More also, co-usage of cold plasma processing with other technologies or as an adjunctive technique ensures effective deactivation of vegetative cells and microbial spores by efficiently ensuring the inactivation of microbial proteins.

**Table 1:** Applications of cold plasma technology in food preservation and shelf-life extension

Food	Operation parameters	Organisms targeted	Microbial Log reduction	Technologies	Gas	Purpose	Reference
**Raw chicken breast meat**	60, 70, or 80 kV for 60, 180, or 300 s	Psychrophiles	> 1.0 log	Dielectric discharge	O_2_ and CO_2_	In-packaging treatment	[64]
**Fresh and frozen pork**	20 kV, 58 kHz, for 0, 30, 60, 90, and 120 s	*Escherichia* *coli O157:H7 and Listeria monocytogenes*	1.5 log and > 1.0 log	Corona discharge plasma jet	Air	Quality Preservation	[65]
**Sprout of rapeseed (*Brassica napus* L.) seeds**	20 kV, 1.5 A, and 58 kH	*Bacillus cereus, Escherichia coli, Salmonella* spp.	1.2–2.2 log	Corona discharge plasma je	Air	Decontamination	[66]
**Ready-to-eat ham**	30 kV at 3.5 kHz	*Listeria monocytogenes*	4 log CFU/cm^2^	Dielectric barrier discharge	O_2_ + N_2_ + CO2 OR CO_2_ + N	Microbial inactivation	[67]
**Cherry**	(40, 60, 80 kV) at (60, 80, 100, 140 s)	*TBC*	3 log	Dielectric barrier discharge	Dry air	Preservation	[68]
**Blueberries**	-	Aerobic mesophilic total viable count, yeasts and molds	0.68–1.25 log CFU/g	Diffuse coplanar surface barrier dis -charge	Nitrogen gas	Microbial inhibition	[69]
**Asian sea bass**	230 V at 50 Hz for 5 min	*Psychrophilic bacteria, lactic acid bacteria, Pseudomonas,* *Enterobacteriaceae* *Clostridium perfringens*		Dielectric barrier discharge	Ar_2_/O_2_	Shelf-life extension	[70]
**Black peppercorns**	10.3 kV and 22.1 min	*Bacillus tequilensis*	3.4 log CFU/g 1.7 log spores/g	Dielectric barrier discharge	Helium	Decontamination	[71]
**Red pepper flakes**	50–1000 W	*Bacillus cereus and Aspergillus flavus*	0.7 ± 0.1 and 1.4 ± 0.3 log spores cm^−2^ 1.5 ± 0.3 and 1.5 ± 0.2 log spores cm^−2^	Microwave plasma	Helium/oxygen (99.8:0.2 v/v)	Preservation	[72]
**Fresh-cut carrot**	100 kV for 5 min (∼250 W power)	Total aerobic mesophiles, and yeast and mold	2 log10 CFU/g	Dielectric barrier discharge	Atmospheric air	Decontamination	[73]
**Ready-to-eat rocket leafy salad**	6 kV, 45 kHz	Total Viable Count (TVC), *Pseudomonas spp*., lactic acid bacteria (LAB), yeasts and molds	0.57 to 1.02 log CFU/g	Dielectric barrier discharge	Atmospheric air	shelf-life extension	[74]
**Powdered Spirulina algae**	10–20 W	*Bacillus subtilis* spore	2 logs	Dielectric barrier discharge	Nitrogen gas	Spore inactivation and preservation	[16]
**Blueberry**	45 kV, 50 s	*Botrytis cinerea* spore	-	Dielectric barrier discharge	-	Preservation	[75]
**Tomato juice**	50 Hz, 3.8 kV, and 40 W	*C. albicans* *and S. cerevisiae,*	2 to 7 log10 CFU/m	AC powered GlidArc reactor	Air	Shelf-life extension	[60]
**Fresh leafy vegetable**	26 kV, 2500 Hz	*Escherichia coli*	5.5 × 103 CFU/mL	Dielectric barrier discharge	Air	Microbial inhibition	[76]
**White shrimp**	10 min, 500 Hz, 40 kV	*Staphylococcus spp., and Salmonella* sp.	1–2 log	Dielectric barrier discharge	Atmospheric air	Preservation	[77]
**Chub mackerel**	60 kV and 60 s	Total viable count	1.47 and 2.7 log CFU	Dielectric barrier discharge	Atmospheric air	Preservation	[78]
**Ready to eat meat (Ham)**	10 kV, 2 kHz	*Salmonella Typhimurium and Listeria monocytogenes*	1.14 log And 1.02 log	Dielectric barrier discharge	N_2_, CO_2_	Decontamination	[79]
**Tomato**	260 V at 50 Hz	*Escherichia coli*	6 log CFU mL^−1^	Dielectric barrier discharge	Atmospheric air	Sanitization	[80]
**Fresh meat**	80 kV for 60, 120 or 300 s	*Brochothrix thermosphacta*	2 Log	Dielectric barrier discharge	CO_2_ + O_2_	Shelf-life extension	[81]
**Radish sprouts**	2.45 GHz, 50–1000 W,	*Salmonella typhimurium*	2.6 ± 0.4 log CFU/g	Microwave generated	Nitrogen gas	Preservation	[82]
**Onion powder**	170 mW m^−2^, 250 mW m^−2^	*Bacillus cereus, Aspergillus brasiliensis and Escherichia coli O157:H7*	2.1 log spores/cm^2^, 1.6 log spores/cm^2^, and 1.9 CFU/cm^2^	Microwave generated	Helium	Decontamination	[83]
**Beef loin**	9 kHz, 8.16, 8.88, 9.44 kV	*Staphylococcus aureus*, *Listeria monocytogenes* and *Escherichia coli*	> 2 log	Dielectric barrier discharge	Air	Preservation	[84]
**Raw chicken breast**	50 W	*Escherichia coli*	1.44 log CFU/g	Plasma jet based on cold arc plasma	N_2_ + O_2_	Decontamination	[85]
**Apple juice**	65 V, 1.1 MHz	*Citrobacter freundii*	5 log	Plasma jet	Argon and oxygen	Decontamination	[86]

### Mechanism of Microbial Inactivation

**Fig. 2:**
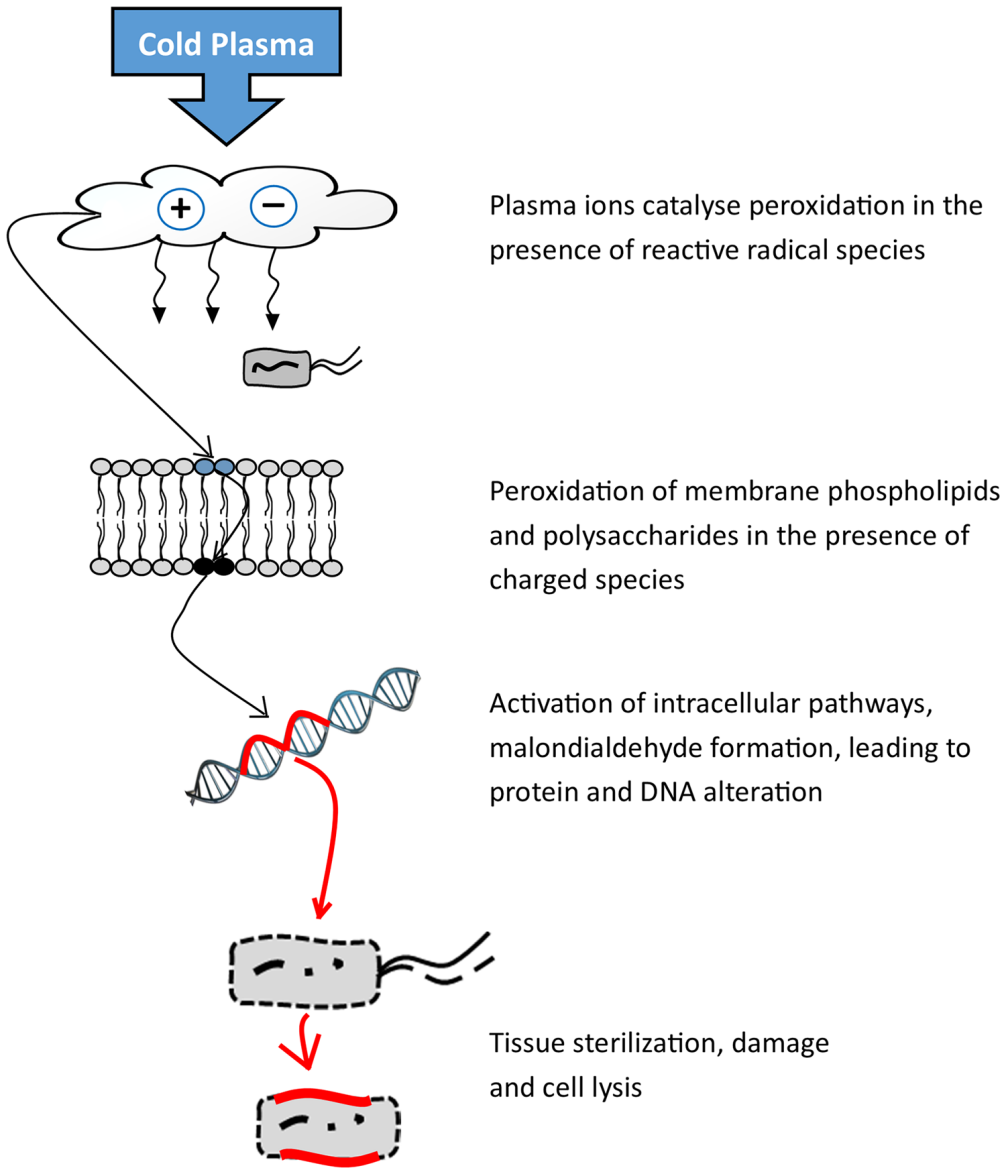
Mechanism of microbial inactivation by cold plasma treatment. Adopted from Dobrynin et al. [88]

The inactivation of bacteria, spores, and other microorganisms in foods and on non-food surfaces has been demonstrated through a variety of studies using atmospheric cold plasma. It has also been reported that cold plasma processing effectively inactivates a broad range of microorganisms by generating reactive species lethal to cells [87]. The process of generating plasma through the oxidation of gases produces reactive species such as reactive oxygen species (ROS), reactive nitrogen species (RNS), UV radiation, energetic ions, and charged particles [88, 89]. However, the antimicrobial effects of cold plasma have been reported to be primarily due to the activities of reactive species [90]. During plasma discharge, the radical species exert antimicrobial effects principally through the induction of oxidative stress, resulting in loss of cellular function and lysis of cell. The induced oxidative stress enhances cell damaged through membrane poration, lipid peroxidation, enzyme inactivation, and DNA cleavage. At the same time, the cold plasma species act on multiple sites of both bacterial and fungal cell, resulting in structural and functional alterations, and ultimately cell death. Likewise, the species cause the chemical breakdown of toxins such as aflatoxin to produce degradation products that are less toxic [51]. An investigation into the roles of plasma species on microbial inactivation found that NO and ions were contributed minimally to the cellular effects, whereas ROS caused rapid bacterial inactivation and induced eukaryotic and prokaryotic oxidative stress [91]. Figure 2 describes the mechanism of free radical-induced oxidative stress resulting in damage and cell lysis. Other plasma byproducts such as UV radiation, H_2_O_2_, and electromagnetic fields acting together enhance microbial inactivation. Treatment of *C. difficile* spores with cold plasma led to ∼3 log reduction in viable spore counts after 5 min [92]. The detection of reactive species responsible for cell death using optical emission spectroscopy demonstrated the presence of atomic oxygen, atomic nitrogen, hydroxyl radicals, nitrite oxide, and nitrate in light emitted from the plasma. In addition, cell membrane damage was observed using scanning electron microscopy, SEM [93]. The SEM showed that CP caused the mycelium fold and collapse depression on *Botrytis cinerea* [75].

### Effect of Cold Plasma on Microbial Spores and Toxins

**Fig. 3:**
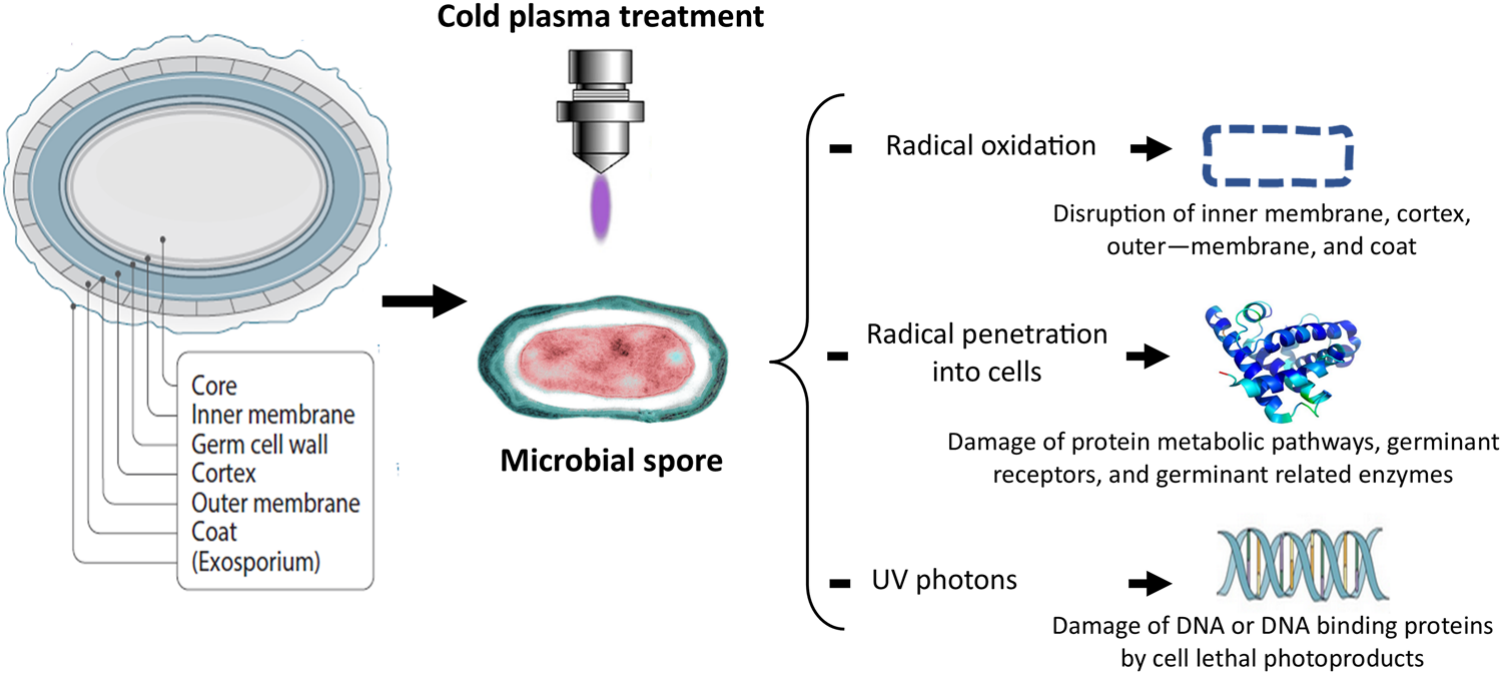
Proposed sites and mechanism of plasma spore inactivation

The effectiveness of cold plasma processing has been extensively investigated on both vegetative microbial cells and inactive spores. Several researchers have demonstrated the efficacy of the technology with arrays of food spoilage and foodborne pathogenic microorganisms including bacteria, and fungi. The effect of cold plasma on resistant spores of *Bacillus* spp., *Geobacillus* spp., and *Penicillium* spp., investigated on food matrix, demonstrated a 3-log_10_ inactivation of *B. coagulans* spores after 10 s [94]. Sporicidal efficacy of cold plasma on *C. difficile* displayed similar results with ∼3 log reduction in viable spore counts after 5 min of treatment [92]. An electron microscopic study demonstrated complete disintegration of fungal spore membrane due to electroporation and etching caused by the reactive plasma species [95]. *B. cereus* and *A. flavus* spores on red pepper flakes were significantly reduced by plasma treatment [72]; whereas, vegetative cells of *G. stearothermophilus* and *B. cereus* spores were effectively eliminated by exposure to cold plasma [96]. Besides the menace of food spoilage and foodborne diseases, toxins produced by certain foodborne microorganisms pose a serious health threat to consumers, resulting to foodborne intoxication. Microbial contamination of food is among the leading cause of hospitalization and death annually. It is estimated that 600 million (about 1 in 10 people in the world) fall ill after eating contaminated food. Microbial toxins mediate wide health consequences, ranging from mild enteric upset to severe and lethal outcomes. Among bacteria involved in foodborne diseases, *Salmonella* spp., *Vibrio parahaemolyticus*, *Vibrio cholerae*, *Staphylococcus aureus*, *Clostridium botulinum*, *Clostridium perfringens*, *Bacillus cereus*, and *Listeria monocytogenes* are considered toxigenic. Similarly, several mycotoxins have been identified in contaminated food and food products, including aflatoxins, ochratoxin A, patulin, fumonisins, zearalenone, and nivalenol/deoxynivalenol. Thus, effective denaturing of microbial toxins ensures food safety. Cold plasma treatment has been evidenced to neutralize microbial toxins and inhibit the synthesis of toxins. High-performance liquid chromatography revealed inactivation of aflatoxin production by *A. parasiticus* and *A. flavus* following plasma treatment [95]. Similarly, a 50% reduction in ochratoxin A content was observed in roasted coffee samples artificially inoculated with the mycotoxigenic fungi (*Aspergillus and Penicillium spp*.), after 30 min of cold plasma treatment [97]. To date, the mechanisms of cold plasma–mediated spore inactivation remains unclear. However, spore shell have been suggested as the primary and main target for a plasma-induced inactivation [94]; neutral reactive oxygen species and UV radiation were also reported to play dominant role in the inactivation of spores [98]. Furthermore, an increases in hydrogen peroxide (H_2_O_2_) concentrations in plasma-treated cells and the increased nitrate (NO_3_^−^) concentrations indicated the role of plasma generated radical ions [61]. The proposed site of action and mechanism of cold plasma inactivation of spores are presented in Fig. 3.

### Effects on Nutritional and Organoleptic Qualities of Food

With the increasing demand for a fresh and healthy product and unaltered nutritional/sensory properties, food preservation entails more than just shelf-life extension. Ensuring that treatment and processes employed for preservation do not adversely affect the properties of the food is of equal significance as a shelf-life extension. Thus, emerging preservation techniques are required to meet the prerequisite of preserving or enhancing the product’s organoleptic properties. Unlike thermal technologies known for altering food products’ innate properties, including nutritional, physical, and textural, cold plasma technology is commended for protecting and preserving food properties. An investigation of the effects of cold plasma technology on the total phenolic content of black pepper grains uncovered that there were no significant differences in the total phenolic content between treated and non-treated samples (*p* > 0.05) [93]. The amino acid species of wine pickled *Bullacta exarata* did not change before and after ACP treatment; however, the contents of serine, glutamine, aspartate, glycine, threonine, and leucine were significantly increased [99]. A similar study reported the absence of significant effects on lipid oxidation, fatty acid composition, and nutritional quality of commercially packaged mackerel fillets treated at 80 kV for extended treatment times of 5 min when compared with untreated control samples [100]. Moreover, high-voltage atmospheric cold plasma treatment increased the yield of phenolic extracts from grape pomace, with high content of anthocyanins and improved antioxidant capacity [101]. In addition, microwave-combined cold plasma treatment had no effect on the antioxidant activity, color, sensory properties, and concentrations of capsaicin and dihydrocapsaicin of red pepper flakes during storage [72]. Furthermore, physiological and metabolomic analysis of cold plasma treated fresh-cut strawberries suggested that plasma treatment improved the biosynthesis of the metabolites in the flavones and flavonol biosynthesis pathway and biosynthesis of phenylpropanoid pathway without altering the textural properties of the fresh-cut strawberries. The study further revealed that plasma treatment enhanced enzyme activities and activated critical gene expression in phenylpropanoid as well as reactive oxygen species metabolism, leading to enhancement of antioxidant capacity and the accumulation of total phenolics, total flavonoid, and anthocyanin [102].

### Effects of Cold Plasma Technology on Biofilm Formation on Food Contact Surfaces

**Fig. 4:**
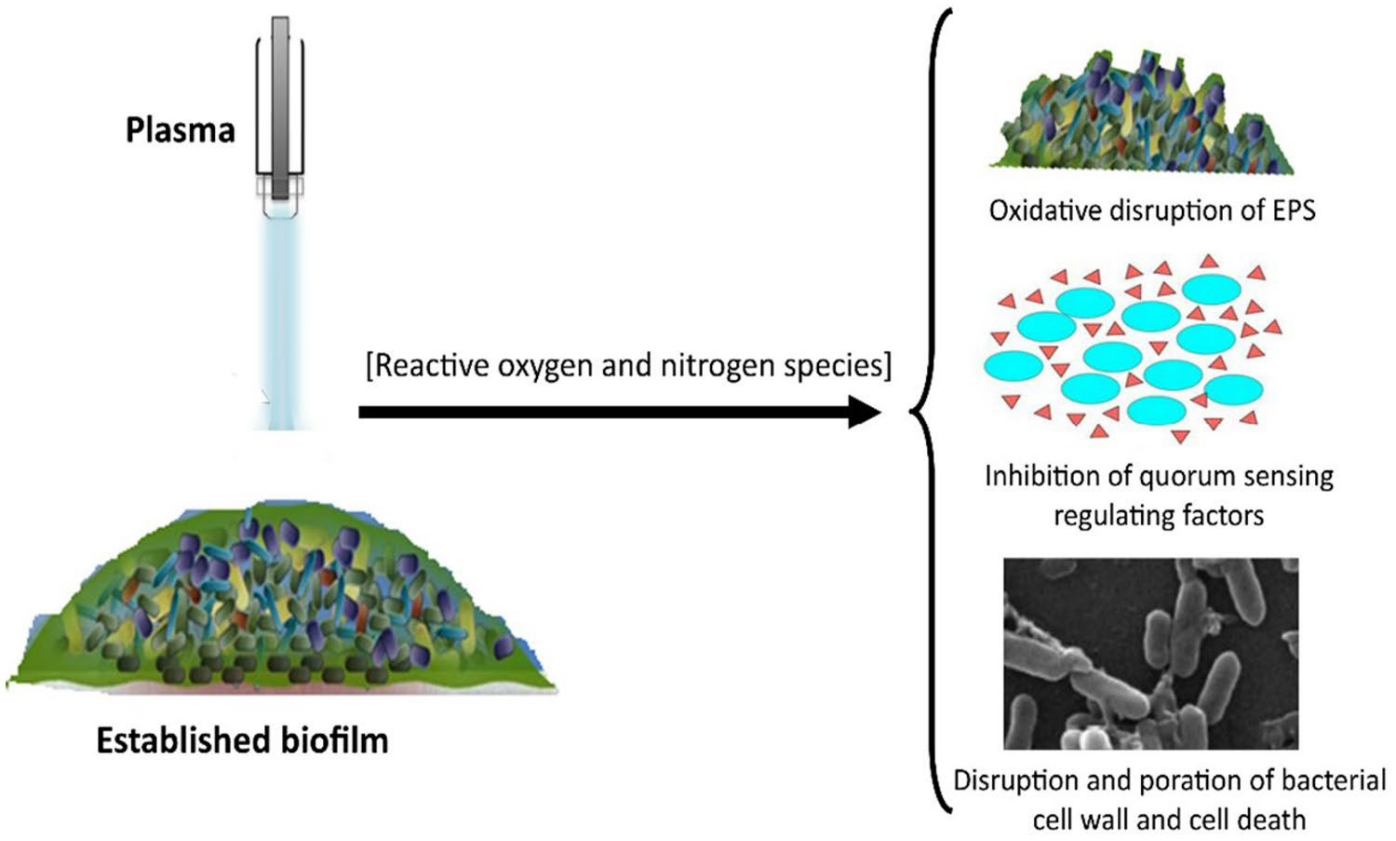
Mechanisms of biofilm disruption by plasma treatment

The formation of biofilm on food contact surfaces is a critical safety concern, due to the cross contamination of products that might arise from disloged biofilm cells. Biofilm formation on contact surfaces is a major microbial survival mechanism and is often associated with resistance to food preservative treatments, sanitizers, and processing. The exopolymeric matrix of biofilms serves as a protective layer that shields the cells from the antimicrobial effects of food-grade sanitizers, antimicrobial chemicals and agents, and food treatment processes. In addition, cells within the biofilm community have been noted to synergistically resist the effects of antimicrobial compounds through the process of quorum sensing. Quorum sensing mechanism enables the community of cells to respond concertedly to changes within their environment, often resulting in the production of neutralizing enzymes, and well as toxins. Several chemical and technological methods have demonstrated excellent activity against planktonic cells but are incapable of inactivating the sessile cells of the biofilm community. This often is associated with the additional protective layer of exopolymeric matrix which inhibits the effective penetration of the antimicrobial agent and hence alters the effective dose or concentration required to inactivate the organism. The biofilm architecture and water channels are often seen as contributors to the slowdown of convective transport and limit diffusion. The use of plasma technology have demonstrated promising effects for the inactivation of biofilms. Exposure of *Staphylococcus aureus* and *Escherichia coli* biofilms to an air‑based atmospheric‑pressure dielectric-barrier discharge plasma for up to 4 min caused approximately 70% and 85% disruption for *S. aureus* and *E. coli* biofilms, respectively [103]. A similar result was reported when atmospheric air plasma technology was applied to inactivate *E. coli* and *Listeria innocua* biofilms. Atmospheric air plasma damaged both the bacterial biofilm cells and its structural integrity. Scanning electron microscopy envinced the disruption of biofilms and pore formation in bacterial cells after exposure to plasma treatment [104]. The elevated reactive oxygen and nitrogen species in bacterial cells treated with atmospheric air plasma demonstrated their primary role in the observed bacterial inactivation process. Penetration of plasma species into samples depends on several factors, including the type of plasma, delivery mode, and the gas composition that makes up the plasma. Active plasma species penetrates into bacterial biofilms, reaching cells through the water channels. Figure 4 presents the proposed mechanisms of biofilm disruption by plasma treatment. A study aimed at investigating the capacity and extent of biofilm penetration by plasma demonstrated that plasma could penetrate a *Porphyromonas gingivalis* 10 days biofilms, of about 30 layers of cells and a thickness of about 15 µm, and effectively deactivate all the bacteria in the 15-µm-thick biofilms [105]. The penetration of cold plasma depends on factors such as the generating gas, intensity, voltage, and the free radical species generated. Liu et al. [106] noted a higher penetration capacity for H_2_O_2_ compared to nitrous/nitric acid and O_3_(aq), even though O_3_ is chemically stable with a much lower concentration than H_2_O_2_ in the gas phase [106]. In addition, oxidative disruption of the exopolymeric matrix by activated plasma species breaksdown the protective shield, exposing the encased cells to the direct effects of plasma. Furthermore, atmospheric cold plasma technology significantly reduced *Pseudomonas aeruginosa* quorum sensing–regulated factors [107] and thus might be a relevant mechanism of biofilm disruption.

### Factors Influencing the Efficacy of Cold Plasma

**Fig. 5:**
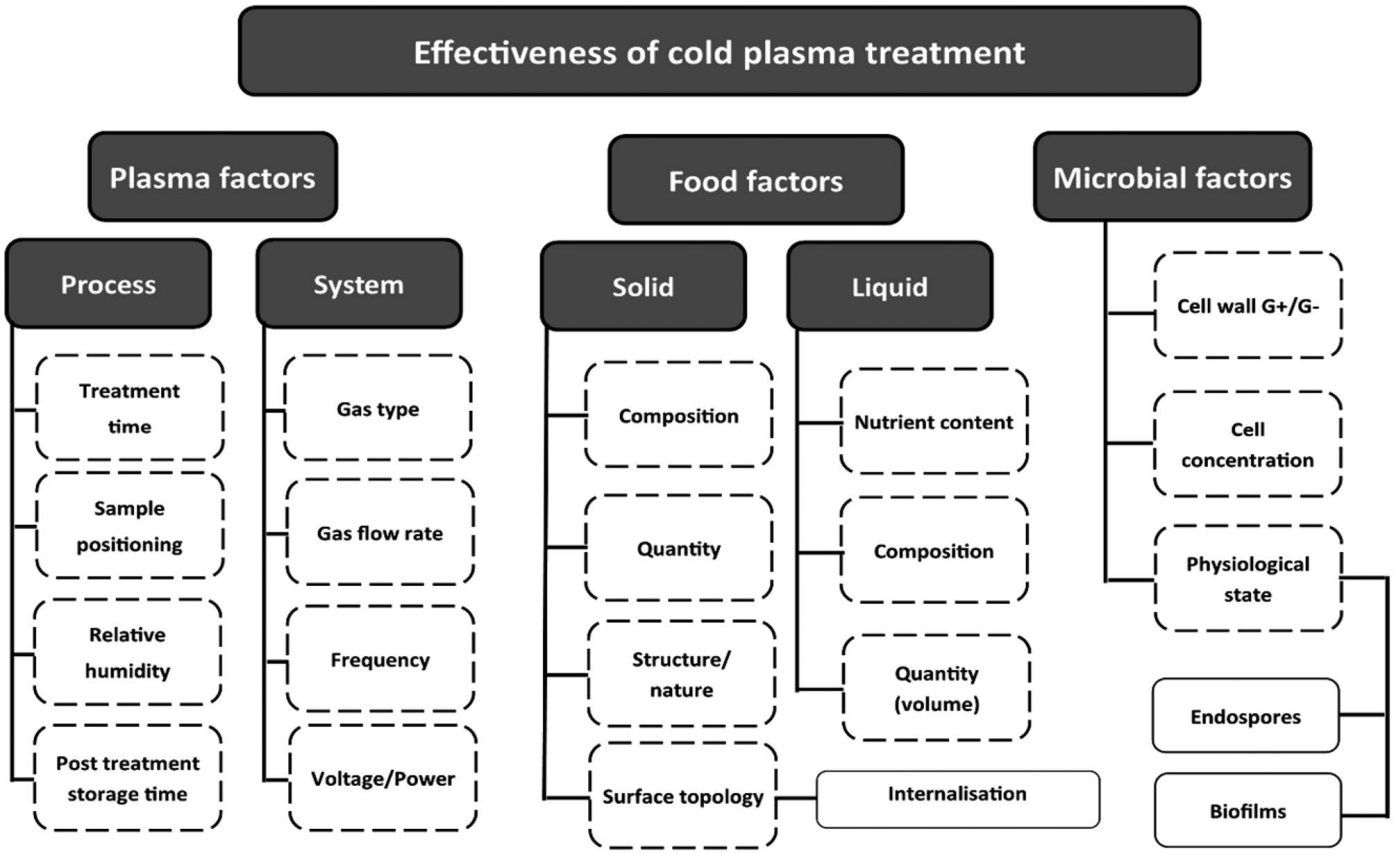
Factors influencing cold plasma treatment. Adopted from Bourke et al. [119]

The efficacy of cold plasma treatment is influenced by several factors, including microbial factors, food factors, and plasma operational parameters. It has been demonstrated that tweaking of instrument setting parameter such as voltage, frequency, treatment time, and working gas composition alters the treatment outcome. A study aiding to investigate the effects of a range of dielectric barrier discharge high voltage atmospheric cold plasma parameters on the inactivation of *Bacillus atrophaeus* spores in a sealed package [108] observed a strong effect of process parameters on the inactivation. Direct plasma exposure for 60 s resulted in ≥ 6 log_10_ cycle reduction of spores in all gas types tested. However, indirect exposure for 60 s resulted in either 2.1 or 6.3 log_10_ cycle reduction of spores depending on gas types used. The authors noted that relative humidity was a critical factor in bacterial spore inactivation by high-voltage atmospheric cold plasma. Several factors regulate the antimicrobial effects of cold plasma treatments [109, 110]. Figure 5 highlights the factors that might affect the outcome of cold plasma treatment. The working parameters and instrumental setting of the cold plasma system are crucial to the effectiveness. An increase in microbial inactivation rates has been reported with variations in process parameters, including power, frequency, and voltage. At higher power 50 W, a higher reduction in the microbial count was achieved at a short time interval; reduction in power required an increased treatment time to achieve an equivalent reduction in microbial count [111]. Similarly, cold plasma treatment on almonds demonstrated an increased reduction in the microbial count after 30-s treatment with increasing voltage [112]. Since plasma can be generated by the excitation of various gases, the type of gas used for plasma generation is crucial to the effectiveness of plasma-mediated microbial inactivation. A comparison of air and nitrogen gas in dielectric barrier discharge plasma for the inactivation of *Campylobacter jejuni* reported that nitrogen gas for 20 s did not yield any reduction (*p* > 0.05) in viable cell count. However, a 0.8-log reduction (*p* < 0.05) in colony-forming units (CFU) was observed when the nitrogen gas was supplemented with 2% (vol/vol) air [113]. Shi et al. [114] noted that relative humidity, gas type, and treatment time are all significant on generated ozone concentration and aflatoxin degradation. The study further reported the generation of a higher ozone concentration in MA65 than in air. In addition, high ozone concentration resulted in the effective degrading of aflatoxin in corn compared with air. Furthermore, working gas mixture and treatment time were observed to exert a strong effect on the atmospheric cold plasma inactivation of *E. coli* and *L. monocytogenes*. Working gas ratios were associated with different bactericidal efficacies. The effectiveness of atmospheric air (gas mix 1), 90% N_2_ + 10% O_2_ (gas mix 2) and 65% O_2_ + 30% CO_2_ + 5%N_2_ (gas mix 3) was found to vary for individual microbial species and strain [115]. The presence of oxygen in plasma generation is critically important for increased microbial inactivation [113]. The enhanced effect demonstrated by oxygen is thought to be due to the generation of ozone (O_3_), a strong oxidizing agent that is used in water treatment as a disinfectant. The dissociation of oxygen molecules at high voltages results in the production of ozone [116]. It has also been observed that treatment time plays a vital role in cold plasma–mediated microbial inhibition [117]. Food intrinsic and extrinsic factors primarily influence the shelf-life and microbial composition of the products. Similarly, factors including nature, type, moisture content, and composition are important to the effectiveness of a food preservation regimen [118].

The antimicrobial efficacy of cold plasma treatment might be influenced by the nature of food constituents (e.g., fat, protein, carbohydrate content). Although several reviews have highlighted the possible effects of food composition and nature on the effectiveness of cold plasma treatment, only a few research has been dedicated to providing clarity on the subject.

The importance of osmotic stress and suboptimal pH on the efficacy of cold plasma inactivation of *Salmonella* Typhimurium and *Listeria monocytogenes* studied on various food structures demonstrated that growth under osmotic stress or at sub-optimal pH promotes microbial cell adaption and resistance to cold plasma treatment [118]. In addition, a study that investigated the influence of surface roughness on the efficacy of cold atmospheric pressure plasma on microbial inactivation showed that an increase in surface roughness resulted in a decreased microbial inactivation efficacy [120]. The water content of food product affects the effectiveness of cold plasma treatment for inactivation of food contaminating microorganisms. The high hydroxyl radicals generated during treatment in liquid water phase promotes effective and increased inactivation of microbial cells [121]. Microbial characteristics, including type, strain, physiological state, growth phase, and mode of existence (planktonic or sensile). Foodborne microorganisms such as *Clostridium* and *Bacillus* species is notable for spore formation and resistance to various food preservation treatments. Spore resistance to preservation treatments can be induced by a number of factors, including structure of the spores, formed with cortex, coat, and exosporangium of the spores; water content in the central region of core of the spore; the saturation of spore DNA by a group of acid-soluble proteins; and low permeability of the inner spore membrane to hydrophilic molecules [122–124]. Microbial exosporium layer protects the interior components of the cell from degradative effects of preservation treatment. Thus, several studies have demonstrated that preservation treatments including cold plasma are usually more effective against vegetative cells. Cold plasma inactivation of *G. stearothermophilus* and *B. cereus* vegetative cells and spores demonstrated a statistically significant difference in the inactivation of *G. stearothermophilus* vegetative cells receiving indirect and direct, as well as for *B. cereus* vegetative cells and spores. However, no statistically significant difference in the inactivation of *G. stearothermophilus* spores receiving indirect or direct exposure [96]. Los et al. [117] reported that the efficacy of cold plasma treatment was strongly affected by the type of microorganism studied. The authors observed high resistance for *B. atrophaeus* endo spores, after direct and indirect plasma treatment for 20 min, and concluded that the endospores of *B. atrophaeus* were considerably more resistant against ACP treatment than the vegetative cells. Furthermore, antimicrobial application in the food industry and processing facilities extend beyond preservation and shelf-life extension. Attachment of microorganisms on food contact surfaces and processing lines is a major cause of food contamination, especially of finished products. Persistent microorganisms in the food-processing environment and resistance to food-grade antimicrobial sanitizers and preservatives have emerged a food safety threat, resulting in finished product recall and foodborne disease outbreak. In addition, the formation of biofilm on food contact surfaces is a leading cause of food contamination, foodborne disease outbreaks, and recall of finished food products. The dense exopolymer matrix of biofilm communities is associated with various undesirable effects in food processing, including impaired heat flow, corrosion of contact surfaces, hampered diffusion of food-grade antimicrobial sanitizers, and resistance to antimicrobials [125, 126]. In recent years, the use of modern green technologies for the decontamination of food processing lines and facilities as well as hospital premises is replacing the conventional use of antimicrobial chemical sanitizers [127–129]. Studies have demonstrated that cold plasma treatment can effectively disrupt and inactivate microbial biofilm. The antimicrobial efficacy of atmospheric cold plasma against *Pseudomonas aeruginosa* biofilms revealed that treatment for 60 s by either the direct or the indirect exposure reduced bacterial populations by an average of 5.4 log cycles from an initial 6.6 log10 CFU/mL. The extension of the treatment time from 60 to 120 s and 300 s reduced biofilms to undetectable levels [130]. Plasma treatment is also indicated for effective disruption of fungi biofilm. The in vitro and in vivo inactivation effects of cold plasma treatment on *C. albicans* biofilm indicated significant inactivation effects [131]. A similar study demonstrated that both gas plasma and plasma activated water treatment decreases of *A. flavus* metabolic activity and spore counts, with maximal reductions of 2.2 and 0.6 log10 units for gas plasma and plasma-activated water, respectively. Biofilm study revealed detrimental effects of gas plasms on biofilm structure [61]. Atmospheric cold plasma treatment on *E. coli* spp., *B. subtilis*, and *Lactobacillus* spp. biofilms resulted in > 3 log10 after 5 min but was ineffective against *B. atrophaeus* [132]. Spores have generally been shown to require extended treatment in other to achieve enhanced inactivation compared with vegetative cells. Ziuzina et al. [133] reported that plasma treatment for 30 s reduced planktonic populations of Salmonella, *L. monocytogenes* and *E. coli* in lettuce broth to undetectable levels. However, depending on storage conditions, bacterial type, and age of biofilm, 300 s of treatment was required to reduce biofilm populations on lettuce by a maximum of 5 log10 CFU/sample. An investigation of the effects of atmospheric cold plasma against microbial biofilms of foodborne pathogenic bacteria demonstrated that plasma treatment for 60 s reduced populations of *E. coli* to undetectable levels, whereas 300 s was necessary to significantly reduce populations of *L. monocytogenes* and *S. aureus* biofilms. The authors, however, suspected possible induction of viable but non-culturable state of bacteria following plasma treatment [107]. A 4-day-old single-species biofilms of *Chromobacterium violaceum* was effective inactivated after 10-min plasma treatment [134]. Moreover, the effectiveness of plasma treatment varies from cell to cell due to variations in cellular properties. Besides spore formation and the formation of biofilms, the thickness of the bacterial cell wall has been correlated to the effectiveness of plasma treatments. Cells with thicker cell walls are more resistant to plasma treatment compared with cells with thin cell walls. Also, the effectiveness of cold plasma treatment is reported to vary between gram-negative and gram-positive bacteria, due to the difference in the cell wall composition [135].

### Advantages and Disadvantages of Cold Plasma Treatment Over Other Technologies

**Fig. 6:**
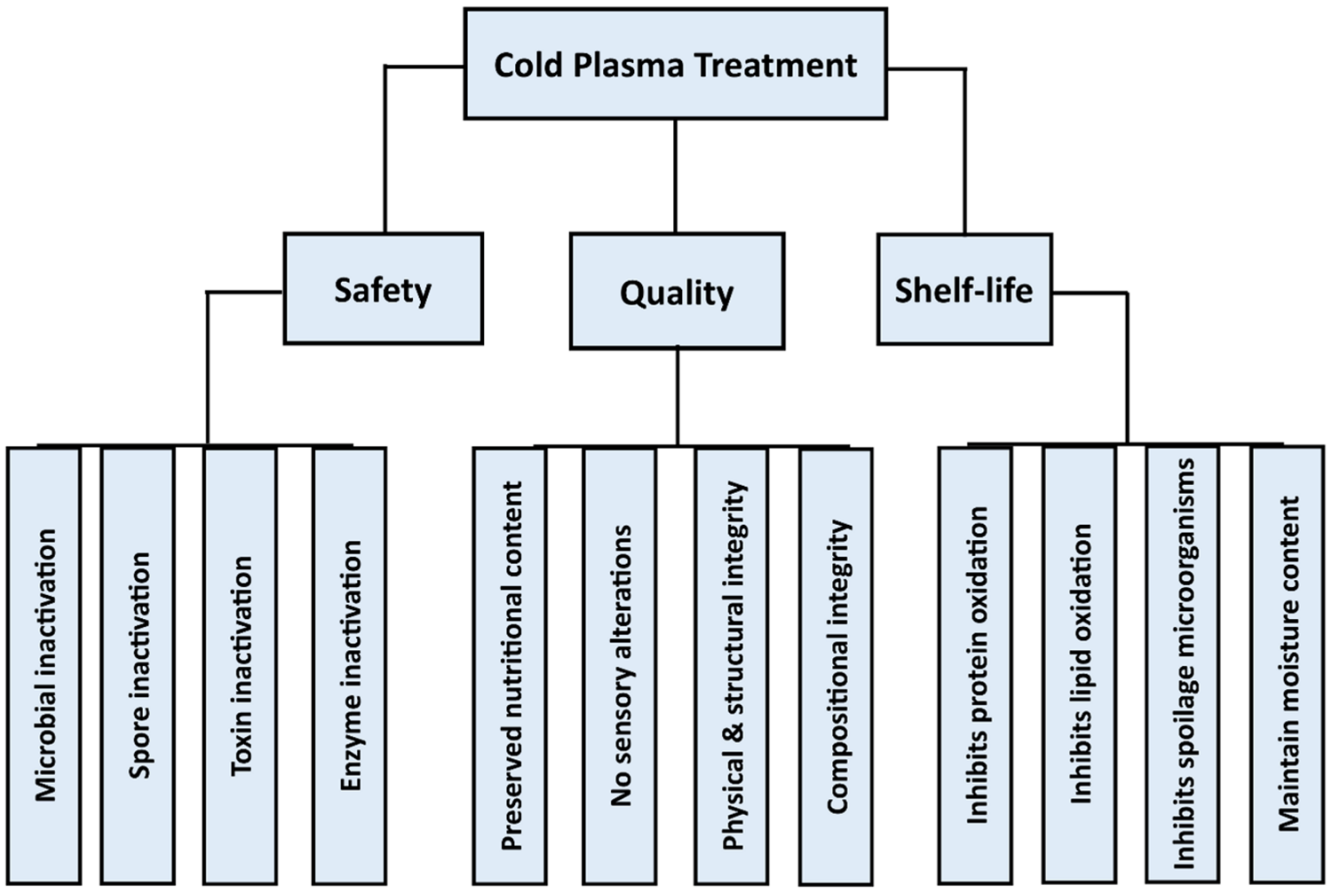
Benefits of cold plasma processing in food preservation

The transition from a chemical preservative-based food processing to a green technology mediated food processing orchestrated by changing consumers taste, safety concerns, and changing food regulations has prompted a proliferation of several technologies seeking application in the food processing industry. Unfortunately, most of the proposed green technology is either limited due to expensive cost of machinery, effect on product quality, not suited for all food type or are inadequate for maximum protection of food product. Cold plasma technique has shown numerous advantages and suitability for the treatment of numerous food types and can ensure adequate inactivation of food contaminating microorganism, including recalcitrant microbial spores, as well as enzymes and toxins. Figure 6 highlights some of the benefits of cold plasma technique in the food industry. In addition, cold atmospheric plasma is versatile with applications in biomedical and food processing and relatively safe. However, in the absence of standardization in terms of treatment parameters and resulting biological effects [136].

Decontamination of food products with uneven surfaces might also pose safety concerns due to the internalization of microorganisms and subsequent compromise of food quality. Furthermore, although cold plasma treatment is assumed to preserve food quality, mild alterations mild lead to unpleasant physical changes that might affect the consumer acceptability and result in economic loss. Chemical changes, such as lipid oxidation, may lead to both economic loss and prompt health concerns [137]. An increase in lipid oxidation was reported in cold plasma–treated mackerel. Similarly, the oleic acid and eicosapentaenoic acid contents were lower in mackerel slices [138]. Thiobarbituric acid reactive substance value showed a time-dependent manner increase in Asian sea bass slices after cold plasma treatment [70].

### Adjunctive Application of Cold Plasma

As new technologies emerge and gain relevance in the food industry, it has become evident that few weaknesses might limit the spectrum of applicability irrespective of the excellent qualities in certain areas. The use of multiple food treatment technologies is perhaps a strategic approach for enhancing food safety. Complimentary usage of food processing technologies at mild or reduced process parameter (for example low temperature and low voltage) augments the effect of individual methods by introducing several hurdles while preserving the sensory, nutritional, and compositional qualities of the food product. This section reviews reported applications of cold plasma technique as an adjunctive or complementary method to promote product safety and shelf-life extension. Cold plasma–based hurdles can be employed to overcome limitations of individual cold plasma treatment, improve the inactivation efficacy, and retain the maximum food quality attributes [139]. The combined effects of ultrasound and cold plasma significantly improved carrot juice quality and safety, with a reduction in total plate count and yeast and mold count compared with products treated with individual technique. Ultrasound and cold plasma treatment enhance carrot juice’s stability with increase, chlorogenic acid, sugar contents, and mineral profile [140]. Microwave-combined cold plasma treatment was effective for inactivating *Bacillus cereus* spores on contaminated red pepper without altering the physical properties. However, process parameters influenced the degree of effectiveness [141]. A similar study reported that microwave-combined cold plasma treatment numbers of *B. cereus* and *A. flavus* spores on red pepper flakes. The antioxidant activity and color of the flakes were unaltered during storage following treatment with either low microwave cold plasma or high microwave cold plasma treatment. Furthermore, low microwave cold plasma also did not affect the sensory properties and the concentrations of capsaicin and dihydrocapsaicin of the flakes [72]. Shiekh et al. [142] employed a combination of pulsed electric field (PEF) pre-treatment, immersion in chamuang leaf extract (CLE) followed by high voltage cold atmospheric plasma for quality preservation and shelf-life extension of *Litopenaeus vannamei*, and reported the lowest microbial load and spoilage bacteria count, and higher likeness scores in PEF treated sample with 2% CLE followed by high voltage cold plasma treatment. They reported synergy between phenolic compounds in CLE and active species generated from high voltage cold atmospheric plasma on the effective inhibition of microbial growth of *L. vannamei* during refrigerated storage. A reduction melanosis scores, lipid oxidation, total volatile base, protein carbonyl contents, and microbial load were attained in CLE and PEF pre-treated shrimp samples exposed to cold plasma compared with control and other treated samples [143]. Application of cold plasma as an adjunctive treatment to nisin pre-treated was effectively employed for the inactivation of *Listeria monocytogenes* of the surface of apples [144]. Simultaneous ultraviolet and cold plasma treatments were used to inactivate indigenous mesophilic aerobic bacteria and *Bacillus tequilensis* spores on black peppercorns without altering the color of the black peppercorns [71]. The versatility of cold plasma and complementarity with other technologies is a principal benefit that can be strategically harnessed for better product quality and shelf-life preservation.

### Cold Plasma for Industrial Application

Part of the challenges associated with cold plasma is the presence of reactive oxygen species (ROS), which triggers lipid oxidation mostly in meat tissue and fish, fatty acids, aldehydes, hydroxyl acids, and keto acid which results in off-flavors and odors during storage [145]. These compounds negatively affect the product’s acceptability and shelf life. In juices, cold plasma technology leads to the degradation of extremely polymerized oligosaccharides [146], which is caused by ozonolysis. The high consumption of gas is also cited as a limitation or drawback of the technology [147], especially for large-scale applications. Furthermore, the high cost of setting up the cold plasma technology tends to be a major challenge or drawback. Moreover, cold plasma treatment is associated with the undesirable alteration of textual properties, acidity, and decolorization of treated foods. Products of animal origin when treated with cold plasma exhibit acceleration of lipid oxidation which has a negative impact on the sensory characteristics. Several opportunities are associated with the use of cold plasma technology. Studies have demonstrated the potential and application of cold plasma technology in the decontamination of wastewater. Cold plasma decontamination lowers the amount of pollutants in wastewater [147]. Furthermore, treatment with cold plasma reduced several water-borne pathogenic microorganisms [46, 148–151] revealed the substantial inactivation of spike protein in plasma inactivated water to inhibit coronavirus transmission. Hence, the combination of CP with other suitable treatments approach possesses viable potential for wastewater remediation in the future.

## Conclusion and Future Directives

In recent past, cold plasma treatment has received increasing popularity in the food industry due to the reported efficiency in microbial inactivation, toxin, enzyme degradation, and mild to absolutely no impact on food properties. Several research and reviews have attempted to provide clarity on the mechanism of microbial spore inactivation by cold plasma technique. This notwithstanding, the detailed mechanism of cold plasma is still not fully elucidated. Herein, the various suggested mechanisms as reported by research articles were explored, which provide direction for further studies. However, as the food production and processing sectors continuously adapt to the changing consumer’s demands and regulations, more technological transition is expected. Non-thermal technologies with less impact on food sensory, flavor, and textural attributes have so far proved beneficial to the food industry, with cold plasma as one of the most recent advances. The effectiveness of plasma treatment in food processing has been demonstrated by several studies on varying types of food products. However, it is relevant to fully understand the mechanism of action of cold plasma treatment, the role of the radical species generated, and to investigate its efficacy across the diverse physiological microbial state. Optimization of process parameters in line with individual food type and product should be a major scientific focus to harmonize parameters and promote usage ease. In addition, there is a need for research to focus on the effects of cold plasma treatment on the compositional, nutritional, and organoleptic properties of food products. Moreover, data on the chemical residual effects and potential toxicity of the various gases used in the generation of plasma would be necessary in guiding decision and regulation. The effects of combined usage of cold plasma with other technologies would be another interesting area for future outlook, and in addition to the evaluation of the residual chemical generation and chemical modification in antimicrobial compounds following plasma exposure. It is also important to verify the claims that treatment of microorganisms with cold plasma could induce a viable but not culturable state.
